# Visuospatial Function in Early Alzheimer’s Disease—The Use of the Visual Object and Space Perception (VOSP) Battery

**DOI:** 10.1371/journal.pone.0068398

**Published:** 2013-07-16

**Authors:** Natália Bezerra Mota Quental, Sonia Maria Dozzi Brucki, Orlando Francisco Amodeo Bueno

**Affiliations:** Department of Psychobiology of the Federal University of São Paulo, São Paulo, São Paulo, Brazil; Federal University of Rio de Janeiro, Brazil

## Abstract

Alzheimer’s disease (AD) is the most frequent cause of dementia. The clinical symptoms of AD begin with impairment of memory and executive function followed by the gradual involvement of other functions, such as language, semantic knowledge, abstract thinking, attention, and visuospatial abilities. Visuospatial function involves the identification of a stimulus and its location and can be impaired at the beginning of AD. The Visual Object and Space Perception (VOSP) battery evaluates visuospatial function, while minimizing the interference of other cognitive functions.

**Objectives:**

To evaluate visuospatial function in early AD patients using the VOSP and determine cutoff scores to differentiate between cognitively healthy individuals and AD patients.

**Methods:**

Thirty-one patients with mild AD and forty-four healthy elderly were evaluated using a neuropsychological battery and the VOSP.

**Results:**

In the VOSP, the AD patients performed more poorly in all subtests examining object perception and in two subtests examining space perception (Number Location and Cube Analysis). The VOSP showed good accuracy and good correlation with tests measuring visuospatial function.

**Conclusion:**

Visuospatial function is impaired in the early stages of AD. The VOSP battery is a sensitive battery test for visuospatial deficits with minimal interference by other cognitive functions.

## Introduction

Dementia is a syndrome characterized by the impairment of cognitive functions, such as memory, language, abstraction, organization, planning, attention, and visuospatial skills [1]. These deficits, which are associated with a decline in the performance of everyday activities, are crucial for the diagnosis of dementia [2]. In general, the course of AD begins with the impairment of memory and executive functions followed by the gradual involvement of other functions, including complex visual disturbance [3], [4].

Visuospatial function in AD can be impaired at the beginning of the disease, declining gradually with the progression of the disease, and can lead to visual agnosia [5]. The visuospatial deficits appear primarily as difficulties with reading, problems in discriminating form and color, an inability to perceive contrast, difficulties in visual spatial orientation and motion detection, agnosia and difficulty in developing visual strategies [6]. These deficits are related to the presence os neuropathology in the visual association cortex [4]. Katz and Rimmer [7] observed numerous plaques and neurofibrillary tangles in the visual association areas in patients without primary visual deficits, which may underlie these deficits. The assessment of these deficits is important in providing more diagnostic information for dementia and new perspectives for intervention.

Visuospatial function involves identification of a stimulus and its location. The tasks of identifying and locating objects activate different cortical areas, such as Brodmann area 5 of the superior parietal lobe, the parieto-occipital junction and the premotor areas [7], [8], [9]. As well as these tasks activate distinct neural circuits that project from the striate cortex and to the occipitotemporal (ventral pathway) and occipitoparietal (dorsal pathway) cortices, respectively [10], [11]. The ventral pathway acts in the visual recognition of objects, whereas the dorsal pathway acts in the recognition of space [12].

Most neuropsychological tests that evaluate visuospatial function require other cognitive skills [13]. For example, the Cubes test (WAIS-III), Rey Complex Figure test, and the clock drawing test require visuoconstructive skills [2], and Hooper’s Test requires analysis and visual synthesis. However, some tests assess only visual orientation and consist of finding objects in space. Some tests involve tasks that assess visual perception and the spatial discrimination of position [8], such as the cancellation tests and the Judgment of Line Orientation test. Among these latter methods is the Visual Object and Space Perception (VOSP) battery [14], [15].

The VOSP battery evaluates space and object perception, and the battery proceeds from the assumption that these perceptions are functionally independent [8]. The subtests require simple responses, and each of them focuses on one component of visual perception, while minimizing the involvement of other cognitive skills [15].

The VOSP battery seems to be sensitive to changes in visuospatial function in various diseases, e.g., posterior cortical atrophy [16] and Lewy body dementia [17]. Additionally, the VOSP has been reported to detect a lack of impairment in visuospatial functions in Huntington’s disease patients [12] and patients with atypical parkinsonian syndromes [18].

Some studies were developed with elderly people and patients with dementia to assess visuospatial function with the VOSP. A survey of healthy elderly using the VOSP battery was conducted in Spain and showed that age was a strong predictor of scores on all subtests, that educational level affected some subtests (Object Decision and Silhouettes), and that gender had no significant effect [19].

In patients with dementia, a portion of the VOSP subtests were used in a study that assessed visuospatial ability in driving. The Incomplete Letters and Cube Analysis tests were used; in both tests, the dementia patients performed significantly worse than controls [20].

In one study comparing the performance of patients with AD, patients with dementia with Lewy bodies and control subjects on the subtests of the VOSP (Screening Test, Incomplete Letters, Silhouettes, Object Decision and Cube Analysis), the patients with early-stage dementia with Lewy bodies showed a significant impairment in visuospatial functions, while only the late-stage AD patients showed impairment [17].

In a longitudinal study in Italy, the VOSP battery was used to evaluate patients with early-stage Alzheimer’s disease; in the first wave, there was no significant impairment of visuospatial functions, although impairments were observed in a second evaluation eight months later on tests of spatial perception [21].

The VOSP has not been used to evaluate AD patients in Brazil. Therefore, there is need for a study to assess visuospatial function in AD and the sensitivity of this instrument in detecting visuospatial deficits in early stages of AD. So, the aims of this study were to evaluate visuospatial function in early AD patients using the VOSP and to determine cutoff scores to differentiate between cognitively healthy individuals and AD patients.

## Methods

### Population Sample

Control subjects agreed to participate in the study by signing an informed consent form. In case of the patients with AD, that could have a compromised capacity to consent, we asked a familiar to fulfill and sign the consent form. The project was approved by the Ethics Committee of the Federal University of São Paulo (No. Of the process: 1924/08) and Santa Marcelina Hospital.

We evaluated 75 participants, including 31 mild AD patients who fulfilled the NINCDS-ADRDA criteria [22]. The exclusion criteria were moderate or severe dementia, uncontrolled systemic diseases, and uncorrected visual or hearing impairments. Patients receiving a stable dose of cholinesterase inhibitors and/or antidepressants for at least two months were included. Patients with AD were recruited at outpatient cognitive units in São Paulo Hospital and Santa Marcelina Hospital.

The control group included 44 healthy elderly (21 women). For these patients, the exclusion criteria were a Mini Mental State Examination [23] score below the median level of schooling (illiterate: 20; 1 to 4 years: 25; 5 to 8 years: 26; 9 to 11 years: 28; more than 11 years: 29) [24]; a Geriatric Depression Scale (GDS) [25] short version score higher than 6; a Functional Activities Questionnaire (FAQ) score greater than or equal to 2 [26]; uncorrected sensory deficits; uncontrolled systemic diseases; neurological or psychiatric disease; and the use of a medication that acts on the central nervous system.

In both groups, the subjects had to have completed more than one year of schooling, been more than 50 years of age, and had no uncorrected visual deficits.

### Neurocognitive Evaluation

The patients were evaluated in a session of about an hour and a half with a battery of neuropsychological tests that assessed cognitive functions and both specifically and in more depth, visuospatial function with the VOSP battery. The project was approved by the Ethics Committee of the Federal University of São Paulo (No. of the process: 1924/08) and Santa Marcelina Hospital.

We evaluated cognitive functions of all participants with the following instruments: Complex Figure Test, perceptual organization and visual memory [27]; Corsi Block-tapping Test, visuospatial short-term memory (direct form) and working memory (inverse form) [28]; Rey Auditory Verbal Learning Test (RAVLT), verbal learning, memory and susceptibility to interference [29]; Verbal Fluency – animal category, spontaneous production of words under restricted conditions [30], [8]; Reduced version of Boston Naming Test – CERAD Neuropsychological Battery, visual naming ability [31]; Cancellation task [32], [33]; Raven’s Progressive Matrices – colored version, a measure of intellectual efficiency and visuoperception [34]; Clock Drawing Test, visuospatial and constructional abilities [35].

### VOSP

Visuospatial abilities were evaluated using the eight-test VOSP battery; four of these tests assess object perception, and four assess space perception. At the beginning, a preliminary test of visual sensory efficiency (Shape Detection) is performed to determine whether the patient has sufficient visual and sensorial capacity to complete the other subtests. All tests are untimed.

In the preliminary Shape Detection test, the patient has to identify whether or not there is a degraded “X” on 20 patterned sheets of paper. One point is given for each correct answer. Subjects with a score of 15 or lower are not able to perform the VOSP battery.

#### Subtests–object perception

- Incomplete Letters: Twenty incomplete letters are shown to the patient, and he is asked to name or identify them. A point is awarded for each correct answer (maximum 20).
- Silhouettes: In this activity, silhouettes (shadows) of animals and objects are shown to the patient, and he is asked to identify them. There are 15 silhouettes of animals and 15 silhouettes of inanimate objects. The boards are arranged in ascending order of difficulty, and the test should be abandoned after five errors. In this study, to determine the cutoff scores, we applied all of the boards, even if the examinee made more than five errors (maximum 30).

Object Decision: Twenty boards with four stimuli are presented. Only one of these stimuli represents something real; the other forms are not defined (distractor stimuli). The patient is asked to identify and name the stimulus that represents the real shape. One point is given for each correct answer (maximum 20).

- Progressive Silhouettes: This test consists of two series in which boards depicting an object are presented; 10 boards depict a gun, and 10 boards show a trumpet. The first board shows the silhouette of the object, and each successive board shows a more complete picture of the object. The patient is asked to identify these two objects with the smallest possible number of boards. The number of stimuli needed to identify these objects is recorded (maximum 20).

#### Subtests–space perception

- Dot Count: The patient is asked to count how many black dots there are on a white card. There are 10 cards. A point is awarded for every correct count (maximum 10).
- Position Discrimination: Ten boards are presented. Each board has two squares with a black dot in the center each. In one of the squares, the point is exactly in the center, while the other point is slightly off-center. The patient is asked to identify in which square the black spot is located exactly in the center. The number of correct answers is recorded (maximum 10).
- Number Location: Ten boards are presented in this test. Each board has two squares arranged one above the other. The top square contains numbers arranged randomly. The bottom square contains only a black dot. The patient is asked to identify which number corresponds to the black dot. Each correct identification earns one point (maximum 10).
- Cube Analysis: Ten boards are presented. Each board features the design of solid structures. The patient is asked to identify how many solids (cubes) there are on each board. The boards are presented in increasing degree of difficulty (maximum 10).

### Statistical Analysis

Data were analyzed using the Statistical Package for the Social Sciences (SPSS) version 17.0. Demographic variables were analyzed from a descriptive point of view. The chi-square test was used to identify possible differences in the sex distribution between the two groups. We used a nonparametric test (Mann-Whitney) to compare the results of neuropsychological tests and to compare the non-continuous variables. The multivariate ANOVA was used to analyze the influence of one variable on the others. We applied the Spearman correlation to investigate the relationship between the subtests of the VOSP and the other neuropsychological tests applied. We used ROC curve analysis to verify which tests best discriminated (sensitivity and specificity) the patients from the controls. The chosen significance level was 5% (p<0.05).

## Results

The demographic and clinical data are shown in Table 1. No difference was observed between the two groups regarding education. However, differences were found in age: patients in the control group were statistically younger than those in the AD group. Given this difference, it was performed a multivariate analysis (multivariate intra-group ANOVA) among the controls and among the patients to evaluate how age influenced the values of the variables of interest. This analysis evaluated each of the variables of interest together and in relation to the variable of age within each group. Only in the control group and only for two variables (the Complex Figure Test Delayed Recall (p<0.001) and Cancellation task (p = 0.019)) did age influence the test scores. Therefore, age was not a major cause of differences between the groups.

**Table 1:** Sample description.

	Diagnosis				
	Controls (n = 44)		Mild AD Patients (n = 31)		Mann-Whitney Test
	Mean (SD)		Mean (SD)		p-value
	(Range)		(Range)		
Age	68.52 (7.06)		74.23 (6.93)		<0.01
	(61–86)		(63–86)		
Education Level	9.59 (4.77)		8.16 (4.16)		0.196
	(3–20)		(3–19)		
Sex	Male	Female	Male	Female	
N	23	21	13	18	p = 0.378*

* χ^2^ test.

All of the participants in the study had scores equal to or less than 6 on the GDS, thereby indicating no symptoms of depression. There was a significant difference between the two groups on the Mini Mental State Examination (p<0.01) and the FAQ (p<0.01). The AD patients had a mean score of 10.65 (SD 4.83) on the functional scale.

The performance of the AD patients and controls on the neuropsychological tests is shown in Table 2. We observed a significant difference between the performance of both groups on all of the tests: the controls outperformed the AD patients with respect to all cognitive skills.

**Table 2:** The scores of the control subjects and AD patients on the neuropsychological assessment tests.

	Controls	Mild AD Patients	
	Mean (SD)	Mean (SD)	p-value *
RAVLT - total	40.91 (8.73)	24.48 (5.87)	<0.001
RAVLT - after interference	8.02 (3.20)	2.19 (2.06)	<0.001
RAVLT - after 30 minutes	7.00 (3.27)	0.90 (1.40)	<0.001
Raven - colored version	26.25 (6.89)	18.52 (5.46)	<0.001
Verbal Fluency - animals	17.00 (4.45)	10.87 (3.71)	<0.001
Rey Complex Figure - copy	32.56 (4.03)	25.95 (8.04)	<0.001
Rey Complex Figure - immediate recall	16.38 (5.96)	5.98 (5.50)	<0.001
Rey Complex Figure - delayed recall	19.49 (7.92)	4.92 (6.42)	<0.001
Clock Drawing test	8.41 (2.11)	6.35 (2.59)	<0.01
Corsi - direct (span)	4.93 (1.13)	3.71 (1.58)	<0.001
Corsi - inverse (span)	4.48 (1.07)	4.15 (1.22)	0.012
Boston Naming (15 items)	14.41 (0.84)	12.19 (1.94)	<0.001
Cancellation task (number correct)	49.82 (8.85)	40.19 (1.29)	0.001
Cancellation task (number of errors)	0.41 (1.04)	2.35 (4.05)	0.009
Cancellation task (time in seconds)	161.27 (60.18)	246.13 (48.42)	<0.001

* Mann-Whitney test.

The comparison of the VOSP scores of the AD patients and the healthy elderly controls is shown in Table 3. All participants completed the Shape Detection screening test properly, making them eligible to continue with the VOSP. Thus, it was observed no significant difference in the performances of the two groups on this first test.

**Table 3:** Comparison between the AD patients and healthy elderly controls in each VOSP subtest.

		Controls	Mild AD Patients		Mann- Whitney
		Mean (SD)	Mean (SD)	p-value	
Screening	Shape Detection	19.50 (0.70)	18.90 (1.33)	0.052	
Object Perception	Incomplete Letters	18.95 (1.31)	16.58 (4.06)	**0.004**	
	Silhouettes	19.31 (5.02)	12.10 (4.55)	**<0.001**	
	Object Decision	15.68 (2.76)	12.52 (3.47)	**<0.001**	
	Progressive Silhouettes	11.27 (2.72)	14.45 (2.84)	**<0.001**	
Space Perception	Dot Counting	9.70 (0.67)	9.48 (0.96)	0.252	
	Position Discrimination	19.23 (1.03)	18.61 (1.73)	0.120	
	Number Location	8.64 (1.70)	6.68 (2.64)	**<0.001**	
	Cube Analysis	8.86 (1.52)	6.55 (2.85)	**<0.001**	

In the object-perception tests, it was found a significant difference in the performance of the two groups on the four subtests, indicating a greater difficulty of patients with AD to perform these activities.

The scores on the space perception tests revealed a significant difference between the groups on the Number Location and Cube Analysis subtests. Conversely, the results of the Dot Counting and Position Discrimination subtests were not significantly different (p = 0.252, p = 0.120, respectively).

We used ROC curve analysis to differentiate the AD patients from the controls using the cutoff points (area under the curve) and the sensitivity and specificity for each variable of interest.

Analyzing the data in Table 4, we could observe the cutoff scores, sensitivity, and specificity for the subtests of the VOSP battery. All of the subtests in which it was observed a statistically significant difference between the AD and control subjects (except Dot Counting and Position Discrimination) showed a good ability to discriminate between the two groups.

**Table 4:** ROC curve analysis for VOSP subtests.

	AUC (95% CI)	Cut-off scores	Sensitivity	Specificity
Incomplete Letters	0.689* (0.566–0.812)	18	88%	42%
Silhouettes	0.859* (0.771–0.947)	16	76%	83%
Object Decision	0.755* (0.648–0.863)	15	66%	68%
Progressive Silhouettes	0.785*(0.679–0.891)	14	71%	77%
Number Location	0.737* (0.622–0.852)	9	63%	74%
Cube Analysis	0.736* (0.614–0.859)	9	75%	68%

* p<0.01.

The Spearman correlation was used to investigate the association between the neuropsychological tests that had visuospatial and perceptive components and the VOSP subtests. The Shape Detection score correlated with the results of the MMSE (r = 0.317), both of which were used as screening tests (Table 5).

**Table 5:** Correlations of the VOSP subtests.

Tests	Spearman Correlation Rho value (N = 75)	Incomplete Letters	Silhouettes	Object Decision	Progressive Silhouettes	Number Location	Cube Analysis
Raven - colored version		0.579*	0.518*	0.494	−0.531*	0.550*	0.600*
Rey Complex Figure - copy		0.445	0.443	0.358	−0.324	0.403	0.483
Clock Drawing Test		0.382	0.526*	0.315	−0.365	0.338	0.406
Corsi - direct (span)		0.405	0.439	0.367	−0.387	0.600*	0.351
Corsi - inverse (span)		0.218	0.352	0.418	−0.422	0.480	0.399
Boston Naming (15 items)		0.535*	0.694*	0.586*	−0.572*	0.516*	0.615*
Cancellation Task (number of correct)		0.368	0.415	0.474	−0.449	0.461	0.429
Cancellation Task (number of errors)		−0.378	−0.218	−0.369	0.193	−0.267	−0.513
Cancellation task (time, seconds)		−0.138	−0.447	−0.303	0.285	−0.241	−0.197

* p<0.01.

All of the subtests that assessed object perception and two space perception subtests (Number Location and Cube Analysis) correlated significantly with the Raven’s test and Boston Naming Test scores. All of these tests require significant visual-perception ability.

The Number Location subtest demonstrated a correlation with the Corsi Block Direct test (r = 0.600); both of these tests require space-perception skill.

## Discussion

In this study, it was assessed visuospatial function in early-stage of AD using the VOSP battery, which aims to assess these functions specifically by eliminating the interference of other cognitive functions.

According to the results, most of the subtests of the VOSP battery effectively differentiated the patients with mild AD from the controls; therefore, we suggest that AD patients visuospatial function is impaired even in the early stages of the disease. This finding corroborates previous studies, noted by Paxton et al. [36], stating that early-stage AD patients often have compromised abilities to draw or copy images and to recognize objects visually in addition to compromised perceptual organization and space perception [13], [37], [38]. However, this finding has been refuted by other studies, which showed impairment of visuospatial functions not in early-stage AD but only in more advanced stages of the disease [17], [21].

It is important to note that the screening test showed sensitivity in discriminating between groups, showing a difference between groups that was close to statistically significant. This finding is important because it shows that in this study we were able to discriminate healthy elderly from AD patients using the Screening test, implying it is a good screening test to aid in diagnosis.

In this study, we observed that some of the subtests of this battery are even more sensible to the initial impairment of visuospatial function in AD, such as the Silhouettes, the Progressive Silhouettes, and the Cube Analysis. Therefore, we recommend the use of these subtests, if the battery cannot be used in its full version.

About the subtests, the results of this study also show that in all tests that evaluated object perception, there was a significant difference between the AD patients and controls. Moreover, this difference was observed in two space perception tests. These variations in the results concerning the perception of objects and space may be related to the fact that distinct neural circuits are responsible for each kind of perception. Visual information is divided into two pathways: the occipito-parietal (dorsal) and the occipito-temporal (ventral). The dorsal pathway processes the information of space ("where") and controls and guides motor activities, whereas the ventral pathway deals with object recognition and perceptual judgment ("what") [10], [11], [12], [16], [39].

According to this, a study that examined two groups of patients with AD, one with and one without visuospatial difficulties, using positron emission tomography (PET) found that the two groups had reduced cerebral metabolism in the parietal and the medial and superior temporal regions compared with controls. However, patients with visuospatial symptoms showed even greater metabolic deficits in the occipital and parietal cortices [40].

Our data are similar to those reported by Lincoln et al. [20], who observed a significant difference in the performance of AD patients and controls on the Incomplete Letters and Cube Analysis subtests. Other studies that used the entire battery observed a significant impairment in the mild AD patients only on the Silhouettes subtest [17], [21].

The subtests of the VOSP battery correlated significantly with the neuropsychological tests, such as the Raven and Boston tests, which are widely used in studies of cognitive evaluation in the elderly [41], [42], [43]. Both tests require an important visual component, which supports our observation that these functions appear to be compromised early in the course of the disease.

The purpose of the VOSP is to assess visuospatial function, while minimizing the involvement of other cognitive functions. As shown here, almost all of the tests that assess visuospatial function require an additional function. We observed that certain subtests require additional knowledge. For example, the Silhouettes and Progressive Silhouettes subtests require semantic knowledge. The Incomplete Letters subtest supposes prior knowledge of the alphabetic letters. Thus, we can observe the interference, however small, of other skills in the VOSP.

In Brazil, no studies have been conducted with this battery. Comparing the preliminary normative data from this population, we observed differences in the Silhouettes and Progressive Silhouettes subtest scores, with the Brazilian scores lower than those observed in the United States and London and closer to those observed in Spain. As we had a sample with a mean of eight years of schooling, we believe that these scores are appropriate without restrictions for this educational level.

Regarding the educational level, we must note that subjects who are illiterate or have little education show significantly lower performance on cognitive assessment tests [44]. Ardila, Roselli and Rosas [45] noted that the performance levels on all evaluated visuospatial tasks differed significantly between illiterate and highly educated subjects.

As in a previous survey conducted in the U.S. and one later in Spain, we observed that the educational level influenced the performance on the Object Decision and Silhouettes subtests [19]. Age also proved to be an important factor that affected space and object perception [19]. Gender had no significant effect. Therefore, we propose the use of the scores obtained in this study for individuals with an age and education level that was compatible with those used in this sample.

Given the above findings, we can say that visuospatial function is impaired in the early stages of Alzheimer’s disease and that the assessment of these functions can provide important diagnostic information.

Future studies must assess larger numbers of AD patients at various stages of the disease to establish a pattern of progression of visuospatial deficit in addition to patients with mild cognitive impairment.

The VOSP battery appears to be effective at assessing visuospatial function and sensitive at detecting visuospatial deficits. We propose that the data reported here be used as preliminary normative data for subjects with similar ages and education levels to the subjects studied.
